# Ethical challenges from a problem-solving intervention with workplace involvement: a qualitative study among employees with common mental disorders, first-line managers, and rehabilitation coordinators

**DOI:** 10.1080/17482631.2024.2308674

**Published:** 2024-02-07

**Authors:** Ida Karlsson, Lars Sandman, Iben Axén, Lydia Kwak, Elisabet Sernbo, Elisabeth Björk Brämberg

**Affiliations:** a Institute of Environmental Medicine, Unit of Intervention and Implementation Research for Worker Health, Karolinska Institutet, Stockholm, Sweden; b Department of Health, Medicine and Caring Sciences, Linköping University, Linköping, Sweden; c Faculty of Social Sciences, Department of Social Work, University of Gothenburg, Gothenburg, Sweden

**Keywords:** Problem-solving, sickness absence, common mental disorders, depression, anxiety, adjustment disorder, ethical challenges, work-directed interventions

## Abstract

**Purpose:**

This study aims to explore ethical challenges potentially arising from a problem-solving intervention with workplace involvement (PSI-WPI) in primary health care (with first-line manager involvement) for employees on sickness absence due to common mental disorders.

**Methods:**

A qualitative design guided by the theoretical framework for systematic identification of ethical aspects of healthcare technologies. Semi-structured interviews were performed with coordinators (*n* = 6), employees (*n* = 13), and first-line managers (*n* = 8). Reflexive thematic analysis was used to analyse and interpret themes.

**Results:**

A main theme was identified “the workplace and healthcare hold different organizational value logics” and four sub-themes: “the PSI-WPI challenged the organizational goals and values of the workplace and healthcare”, “the PSI-WPI challenged organizational values on fairness”, “the PSI-WPI challenged the professional roles of first-line managers and rehabilitation coordinators” and “the PSI-WPI introduced a need for the employee to juggle the employee and patient roles”.

**Conclusion:**

Different organizational value logics, values, and goals can introduce ethical challenges. We advise clarifying stakeholders’ roles and preparing employees and managers for the return to work process by providing sufficient information. The ethical challenges and suggested measures to minimize them, should be considered when planning return to work interventions that involve several stakeholders.

## Introduction

Common mental disorders, i.e., mild- to moderate depression, anxiety, adjustment disorder, and stress-related disorder, are among the leading causes of sickness absence, internationally and in Sweden (OECD, 2021; World Health Organization, 2017). Employees suffering from common mental disorders are more often absent from work and absent for longer periods compared to employees on sickness absence for other reasons (Mishima et al., 2020; OECD, 2018). In Sweden, psychiatric conditions, including common mental disorders, cause approximately half of all ongoing sickness absence cases (The Social Insurance Agency, 2020). Common mental disorders cause individual suffering, economic loss, and reduced work ability (OECD, 2018). Additionally, employers face increased costs due to productivity loss, the healthcare sector faces an increased burden of individuals in need of care, and society faces high costs from social insurance benefits (OECD, 2018, 2021), all of which translates to costs of around €600 billion per year in Europe (OECD, 2018).

Over the past decade problem-solving interventions in combination with workplace involvement (PSI-WPI) such as including the manager in the return to work process, have been evaluated (Arends et al., 2012; Axén et al., 2020; Dewa et al., 2015; Keus van de Poll et al., 2020; Nieuwenhuijsen et al., 2020; van Oostrom et al., 2010) and this combination can lead to a decrease in sickness absence days during the first year among employees with depression (Nieuwenhuijsen et al., 2020), decreased time until first return to work among employees on sickness absence due to common mental disorders (Axén et al., 2020) and enhanced partial return to work within the first year among individuals on sickness absence due to adjustment disorders (Arends et al., 2012). But evidence whether long-lasting effects (>12 months) of PSI-WPI include a reduction of sickness absence and full return to work are inconclusive (Axén et al., 2020; Dewa et al., 2015). Studies that have evaluated PSI-WPI have mainly been conducted in occupational health services (Arends et al., 2014; Keus van de Poll et al., 2020; van Oostrom et al., 2010). Hence, these interventions have been provided by occupational health service specialists and evaluated in settings with a tradition of work-directed interventions (Arends et al., 2014; Keus van de Poll et al., 2020; van Oostrom et al., 2010). However, the access to occupational health services differs in different countries and sectors and usually, only larger companies (>500 employee) have access (Rantanen et al., 2017). In Sweden, access to occupational health services is regulated by law (Ministry of Labour, 1977) despite this, only around 60% of employees have access (The Swedish Work Environment Agency, 2022).

The Swedish primary health care system is managed by the regional councils, provides services to all citizens and is the first-line psychiatry treatment for persons with common mental disorders (Sundquist et al., 2017). There are publicly and privately funded primary health care centres although the majority are tax funded (The National Board of Health and Welfare, 2019). The Swedish healthcare system is built on norms of providing care based on need and autonomy, solidarity, equality, and justice. The Swedish Health and Medical Care Act regulates how healthcare should be organized and states that healthcare should be available for all citizens, with priority given to those with greatest need (Ministry of Social Affairs, 2017). Although the evidence so far on work-directed interventions on reducing sickness absence is promising, primary health care has a limited history of providing such interventions.

To test the effectiveness of problem-solving in primary health care, the PROSA randomized controlled trial was conducted. PROSA consisted of a PSI-WPI for employees on sickness absence due to common mental disorders in primary health care in Sweden (Björk Brämberg et al., 2018). PSI-WPI was provided as an individual-level intervention with collaboration between the individual’s workplace and healthcare. In PROSA, PSI-WPI is based on problem-solving principles, guided by the employee’s own ideas (Nezu & Nezu, 2018). The method involves the employee’s first-line manager and is provided by rehabilitation coordinators. The PSI-WPI holds assumptions on three stakeholders. First, on the employee to take part in the problem-solving process by identifying problems, think about and implement solutions, agree to manager involvement, and participate in a three-party meeting. Second, on the first-line manager, to participate in a problem inventory, a three-party meeting, and arrange work accommodations. Third, on the rehabilitation coordinators to educate and support the employee in the problem-solving process and coordinate the collaboration between the employee and first-line manager. A recent publication explored experiences, facilitators of and barriers to participate in PSI-WPI which showed that the intervention was experienced as supportive and enabling a dialogue but was also considered demanding and good relationships were needed (Karlsson et al., 2023). For a more detailed description of the problem-solving process, see PROSA study protocol (Björk Brämberg et al., 2018).

Current research recommends facilitating the return to work process by adopting a multi-disciplinary approach with communication among relevant stakeholders (Corbière et al., 2020). When introducing work-directed interventions such as a PSI-WPI in primary health care, a collaboration among the employee, the workplace, and healthcare is introduced that goes beyond the usual encounter between a patient and health care professional (Ejeby et al., 2014; Finnes et al., 2019; Nieuwenhuijsen et al., 2020; Salomonsson et al., 2017). In occupational health services, first contact is initiated by the employer or the employee (who would usually first need approval from their employer to initiate the contact). Through agreements, the employee might be able to request support from the occupational health services without informing the employer. In contrast, in primary health care, contact is usually initiated by the employee, and the employer becomes involved only if the employee consents. In the primary health care system, the employee is protected by the Patient Act (Ministry of Social Affairs, 2014) and Publicity and Privacy Act (Ministry of Social Affairs, 2009) and sharing of information outside healthcare would require consent from the employee. Because of these differences in approaches, collaboration between stakeholders that represent different organizations, such as in the PSI-WPI, may result in ethical challenges (Heintz et al., 2015; Iavicoli et al., 2018; Westerholm, 2007). For example, different stakeholders might have different ideas about decision-making authority, goals for the individual (Young et al., 2005), work ability, health, and illness (Borrell-Carrió et al., 2004). A scoping review has described the roles and actions of the stakeholders involved in the return to work process similarly to the stakeholders’ roles during the PROSA trial (Corbière et al., 2020). That review further highlighted the importance of regular contact among stakeholders, understanding the role of each stakeholder, and good communication and exchange of knowledge among stakeholders (Corbière et al., 2020).

The literature on return to work has reported challenges related to specific situations in healthcare, the workplace, and the individual that reflect potential ethical challenges (Holmlund et al., 2022; Iavicoli et al., 2018; Müssener et al., 2015; Porter et al., 2019; Toth & Dewa, 2014; Toth et al., 2021). In healthcare, prioritizing certain types of patients can be an ethical challenge (Holmlund et al., 2022) and negative encounters with healthcare professionals can have negative effects on employee’s return to work process (Müssener et al., 2015). At the workplace, ethical challenges include conflicts between the organization’s economic performance and adapting the work to support the employee (which can reduce overall performance) (Iavicoli et al., 2018). On the individual level, disclosure has been described as a complex decision process in which the employee needs to learn about the relevant processes and then balance the risks and benefits of sharing sensitive information with for example a manager or colleagues (Toth & Dewa, 2014; Toth et al., 2021). Though managers reportedly preferred that an employee disclose illness, problems, or needs (Porter et al., 2019) the employee risks being treated differently, that confidentiality will not be respected, or stigmatization (Toth et al., 2021). A primary reason to disclose information about illness is if the work is affected by current symptoms, and an expected benefit is to receive understanding and accommodated work tasks (Toth & Dewa, 2014; Toth et al., 2021). To support the employee in their decision making about disclosure, it is beneficial to have a preparatory dialogue between the employee and rehabilitation coordinator and a three-party meeting with the employee, first-line manager and rehabilitation coordinator (Holmlund et al., 2022). Stigmatization (i.e., treating someone differently or unfairly due to their mental illness) of individuals with mental health problems from managers and/or colleagues can hinder return to work (Toth et al., 2021). Employees have reported that managers’ lack of understanding of, and interest in their situation without acknowledging problems at work, further hinder return to work (Joosen et al., 2022). In addition, stigmatization has also been reported from health care providers towards patients with mental health problems, which can hinder access to care and affect the quality of care (Knaak et al., 2017).

To date, there have been few evaluations of ethical challenges in work-directed interventions. The strong push for evidence-based healthcare has resulted in demands for health technology assessment when introducing new interventions. Despite the term health technology, health technology assessments are generally supposed to cover also other forms of interventions within the healthcare and social sectors with necessary adaptations. An integral part of health technology assessment is to analyse interventions from an ethical perspective (van der Wilt et al., 2022). To do this several generic frameworks exist, but also country-specific adaptations of frameworks where ethical-legal values and norms of the country in question are analysed. By using a country-specific framework, the systematic identification of ethical aspects which aims to identify and describe ethical challenges that can arise when introducing new interventions in healthcare contexts and across organizations, will be well adapted to the context where the intervention is supposed to be used (Heintz et al., 2015). The current analysis focuses on potential ethical challenges of involving the workplace and first-line manager in the return to work process, and on the potential challenges faced during the collaboration between the workplace and healthcare. Knowledge gained from exploring ethical challenges arising from a PSI-WPI in primary health care in Sweden can be used to support the adaptation of the intervention before a large-scale roll-out. Further, the identification of ethical challenges could serve as a guide for stakeholders on how to handle or minimize ethical challenges when providing work-directed interventions with multiple stakeholders and organizations.

## Aim

To explore ethical challenges potentially arising from a PSI-WPI in primary health care (with first-line manager involvement) for employees on sickness absence due to common mental disorders.

## Method

### Design

A qualitative study design was used to explore potential ethical challenges from the perspectives of employees, first-line managers and rehabilitation coordinators, guided by the framework for systematic identification of ethical aspects of healthcare technologies (Heintz et al., 2015). The reporting of the study follows the consolidated criteria for reporting qualitative research (Tong et al., 2007).

### Theoretical framework

The framework for systematic identification of ethical aspects by Heintz et al (Heintz et al., 2015). contains four domains of which we explored the first three: the expected effect from the intervention on health, the interventions’ accordance with ethical norms, and structural factors that may bring ethical challenges (the fourth domain concerning long-term ethical consequences was not included as this would require a long-term follow-up) (Heintz et al., 2015).

### Setting

The PROSA trial was conducted in Swedish primary health care. It was provided by rehabilitation coordinators (representing healthcare) to employees on sickness absence due to common mental disorders and involved the employee’s first-line manager (representing the workplace). In Sweden, the role of the rehabilitation coordinator is to provide support to individuals on sickness absence and coordinate stakeholders involved during the return to work process (Ministry of Social Affairs, 2019; Swedish Association of Local Authorities and Regions, 2021). This coordination might involve contact with the first-line manager, if such is agreed upon with the employee (Swedish Association of Local Authorities and Regions, 2021). Several laws are in place to protect the patient, regulate the healthcare delivered, and ensure safe work environments. The Patient Act holds that healthcare should strengthen and clarify the patient’s position in regard to integrity, autonomy, and participation (Ministry of Social Affairs, 2014). At the workplace, the employer is responsible for providing a safe and healthy work environment to prevent illness or accidents, which is regulated by the Work Environment Act (Ministry of Labour, 1977) and the Swedish Work Environment Authority regulations on Systematic Work Environment Management (The Swedish Work Environment Authority, 2001). The employer is responsible for the rehabilitation of employees on sickness absence and, if necessary, for providing accommodated work tasks, or for adapting the employees’ work hours or physical work environment (The Swedish Work Environment Authority, 2015, 2020). In Sweden, individuals experiencing disease or injury that effects their ability to work are entitled to sickness absence benefits, regulated through the Social Security Law (Ministry of Social Affairs, 2010). For individuals who are gainfully employed, the first two weeks of sickness absence (except one qualification day) is paid by the employer and if a longer duration sickness absence is needed, benefits are paid by the Swedish Social Insurance Agency (The Social Insurance Agency, 2022).

## Procedure

### Sampling

All rehabilitation coordinators from the intervention group (*n* = 9) were asked to participate in an individual interview, six rehabilitation coordinators agreed to participate. Among employees from the PSI-WPI group and their first-line managers, a strategic sampling procedure based on gender, age and work sector was applied. All employees from the intervention group who had completed the 12-month follow-up by May 2020 (*n* = 72) were contacted by phone with a request to participate in an individual interview. Because of recruitment difficulties mostly due to employees not responding to the invitation to the interview (*n* = 39) or employees declining to participate in an individual interview (*n* = 20), the final sample resulted in 13 individual interviews. Employees who answered the telephone call from the research team were asked if the research team could contact their first-line manager for an interview. Contact information was provided for 16 first-line managers, of which eight agreed to participate in an interview.

### Data collection

Data was collected by semi-structured interviews developed from an interview guide first developed by Holmlund et al (Holmlund et al., 2022). The open-ended questions evaluated ethical challenges related to autonomy, privacy, equality, justice, third party and professional roles in relation to the components included in PSI-WPI. The interviews contained questions on two parts (1. Exploring experiences, facilitators of, and barriers to participate in PSI-WPI, 2. Ethical aspects of participating in PSI-WPI) in which the second part of the interview was used for this study. Three separate guides were designed, one for each stakeholder [Appendix 1, Interview guides]. The interviews were conducted from April until October 2020 (rehabilitation coordinators in April, employees from April to September, first-line managers from May to October). The interviews with rehabilitation coordinators took place at their workplaces; employees and first-line managers were interviewed over the phone due to the COVID-19 pandemic. Interviews were conducted with a total of 27 participants (13 employees, eight first-line managers, and six rehabilitation coordinators). The mean length of the interviews was 29 minutes (range 16–40) for employees, 25 minutes (range 18–35) for first-line managers, and 56 minutes (range 39–67) for rehabilitation coordinators. All interviews were recorded digitally, transcribed verbatim, and cross-checked for accuracy. Identifying information was removed from the transcripts before the interviews were analysed. Author IK, a registered nurse and doctoral student, conducted the interviews with first-line managers and four employees. Nine employees and the rehabilitation coordinators were interviewed by author ES, who has a doctoral degree in social work. Both interviewers were female, experienced interviewers, and had knowledge in qualitative methods. All participants were informed that the interviewers were not part of the planning or execution of the PROSA trial. The interviewers had no previous relationships with the participants.

### Participants

The characteristics of the study participants are given in Table I. At inclusion, employees were on sickness absence due to mild- to moderate depression, anxiety disorder, or adjustment disorder for a minimum of 2 weeks and up to a maximum of 12 weeks. The employees in this study showed no significant differences at baseline regarding education, age, exhaustion, depression, or anxiety when compared to all PROSA study participants who answered the baseline questionnaire (*n* = 172).

**Table I.** Participant characteristics.

		Employees	First-line managers	RCs
		n = 13	n = 8	n = 6
***Age in years***	***Mean (range)***	44 (22–60)	46 (34–61)	59 (39–68)
*Gender*	*Female*	13	4	6
	*Male*		4	
*Education*	*Up. Sec. School*	8	1	
	*University*	5	7	6
*Rehabilitation coordinator basic profession*	*Nurse*			4
	*Occupational therapist*			1
	*Physiotherapist*			1
*Work sector*	*Public sector*	8	4	6
	*Private sector*	5	4	
*Sick leave at 12 months follow-up*	*Returned to work*	10		
	*On sick leave*	3		
*First-line managers employee responsibility*	*<10 employees*		2	
	*10–20 employees*		4	
	*>20 employees*		2	

### Data analysis

The interviews were analysed by theoretically driven reflexive thematic analysis (Braun & Clarke, 2006, 2021). The theoretical framework for systematic identification of ethical aspects of healthcare technologies (Heintz et al., 2015) formed a theoretical base on which a coding scheme was built including the following ethical values: autonomy (can the employee actively participate in the intervention and take part in decision making?), privacy (can privacy be maintained during information sharing between stakeholders?), equality (is there a risk of unequal treatment of similar patients?), justice (is there a risk of an unjust division of healthcare’s resources?), effects on third parties (can the intervention have an impact on the employee’s colleagues or workplace?) and professional values (can professional values influence delivery?). As recommended by Heintz et al (Heintz et al., 2015). a brainstorming session about potential ethical challenges faced by the involved stakeholders was performed by authors IK, ES, LS, and EBB before starting the analysis. The discussion included potential challenges of maintaining confidentiality, an expectation of decision-making capacity, and equal and just access to the intervention.

The thematic analysis was conducted in the following steps. 1) Familiarisation: IK listened to the interviews and IK, ES, LS, and EBB read the transcripts to get an overall understanding of the content. 2) Systematic data coding: IK and ES, supervised by LS and EBB, data was coded deductively by underlining, and categorizing relevant codes, i.e., those close to an ethical value in the coding scheme, using the software Nvivo (released in March 2020). 3) Generating initial themes: an inductive approach was taken for interpreting the latent content of the deductively organized coded data i.e., forming preliminary themes from the codes derived from the previous steps. IK, ES, LS, and EBB formulated seven preliminary themes (privacy, identity, autonomy, professional ethos, third party, equality, justice), each describing an ethical challenge. All authors discussed the preliminary themes. 4) Developing and reviewing themes: IK, LS, and EBB had several meetings to review and revise the initial themes. During these meetings, a process of discussion and reflection was implemented by going back and forth between data, codes, and themes. Themes were reviewed and refined by assessing coherence and heterogeneity. During this revision, a main theme was developed describing an overarching ethical challenge, that could be framed in four sub-themes. Of the seven initial preliminary themes, three pairs were combined (equality and justice, privacy and identity, and autonomy and professional ethos) to ensure heterogeneity among the four resulting sub-themes. 5) Refining, defining, and naming themes: the content, definitions, and naming of the main theme and sub-themes were discussed and approved by all authors. 6) Writing the report: IK, LS and EBB wrote the report. All authors read, reviewed, and contributed to the final report.

## Results

**Figure 1.**
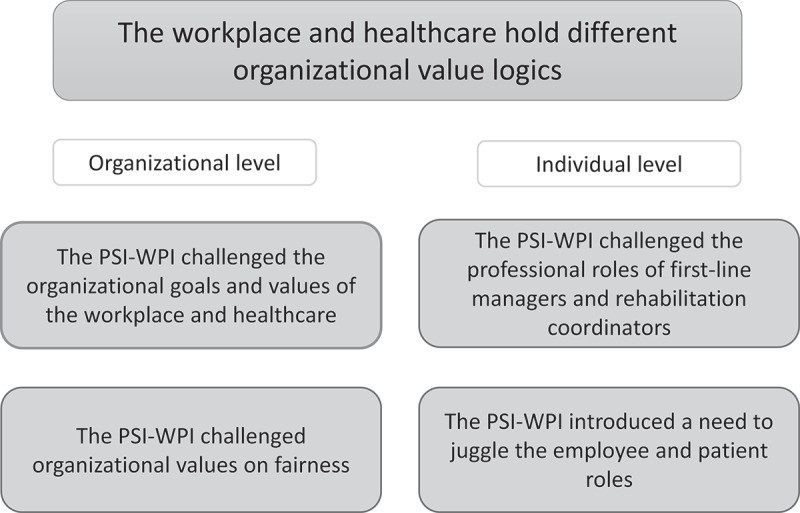
An overview of the main theme and four sub-themes.

The results are presented as a main theme with four sub-themes, two on the organizational level and two on the individual level (Figure 1).

*PSI-WPI, problem-solving intervention with workplace involvement.

### Main theme: The workplace and healthcare hold different organisational value logics

The analysis identified different value logics (i.e., the overall structure and content of goals, values, and norms in organizations) between the workplace and healthcare. At the workplace, the goals were mainly related to productivity and people were rewarded according to their work effort. In the healthcare organization, the overarching goal was to promote health, primarily related to patients’ needs. Since the PSI-WPI involved the workplace and healthcare, the value logics implied an interplay but also a potential conflict between the two organizations’ goals and values.

In ordinary circumstances, the two organizations held separate mandates and responsibilities as well as separate legal- and ethical frameworks. For example the workplace was regulated by the Work Environment act (Ministry of Labour, 1977) and healthcare was regulated by the Health and Medical Care act (Ministry of Social Affairs, 2017). During the PSI-WPI, first-line managers and rehabilitation coordinators, representing the two organizations, were encouraged to collaborate towards the common goal of helping an employee on sickness absence successfully return to work. Though the first-line manager was responsible for providing systematic work environment management, the PSI-WPI also meant that the first-line manager always was expected to participate in a three-party meeting and often received health-related information. This could be viewed as going beyond both the workplace’s and health care’s usual mandates and responsibilities. This way of collaborating may have resulted in ethical challenges on the organizational level relating to organizational goals, effects on third parties (i.e., the sick-listed employee’s colleagues) and organizational values about fairness. On the individual level (employee, first-line manager, and rehabilitation coordinator), the collaboration may have resulted in ethical challenges relating to the professional roles of the first-line managers and rehabilitation coordinators, sharing of information among stakeholders, employee autonomy, and the employee/patient role (see Figure 1 for an overview).

### Sub-theme: The PSI-WPI challenged the organisational goals and values of the workplace and healthcare

The PSI-WPI introduced a collaboration between the workplace and healthcare which required them both to go beyond their organizational goals and values.

The workplace’s goal was to maintain the productivity and efficiency of the organization while ensuring a healthy work environment. Values mainly related to productive work and a distribution of resources were based on work effort. Healthcare’s goal was to support individuals’ health needs. Values related to supporting patient autonomy and integrity, and resources were distributed based on need. The workplace participation (through the first-line manager) in the PSI-WPI meant that they, to a large extent, received information about their employees’ health problems and rehabilitative treatment from primary health care when participating in three-party meetings. To some extent, these participating first-line managers received more information about the employees’ common mental disorder problems than they would have, in the usual circumstances surrounding sickness absence (normally there would be a narrower focus on an employee’s current health problems as a part of their work ability). One first-line manager explained: “*Our interest is to get the employee back into production as soon as possible. If this can be helped by us having a three-party meeting, then we are absolutely positive. Because I mean, my main task as first-line manager is to ensure that my employees are healthy and able to do their job”* (First-line manager 1). In contrast, health care’s participation in the PSI-WPI (through the rehabilitation coordinator) meant that healthcare to some extent needed to specifically consider work-related problems, at least more than healthcare usually would. Balancing the needs of the first-line manager and the rehabilitation coordinator meant that a shift in perspectives was necessary, the workplace needed to consider health-related factors and healthcare needed to consider work ability.

In addition, the PSI-WPI may have had effects on third parties at the workplace, which in turn could have affected the organization’s goals. The first-line manager faced the potentially conflicting goals of catering to an employees’ rehabilitation needs by arranging work accommodations, versus the organization’s productivity goals and the concerns of other employees. If the employee on sickness absence received work accommodations, such as fewer assignments or less responsibility, the overall productivity may have been affected, and other employees may have had to increase their own productivity. One first-line manager said, “*If the employee says no, right now I just want to work these hours, then you have to have a discussion about that, it might not be possible to only work these hours because there are colleagues too, and it can´t create a poor situation for a colleague. It is also the work environment for the colleagues if you know what I mean, the colleagues of the employee”* (First-line manager 2). As described in the quotation, the manager highlighted his/her need to consider the work environment for the employee on sickness absence as well as the colleagues of that employee, ensuring that a sufficient work accommodation for one employee did not become a liability for his/her colleagues.

### Sub-theme: The PSI-WPI challenged organisational values on fairness

The different organizational value logics influenced what was understood as a fair distribution of resources within the workplace and healthcare.

A potential challenge of introducing the PSI-WPI was that it may have impacted the distribution of resources within the workplace and healthcare. At the workplace, first-line managers may have faced decreased productivity and increased demands on other colleagues during a sick-listed employees return to work, with neither the workplace nor that employee´s colleagues receiving extra compensation. This situation may have been viewed as unfair given the goal of productivity. For healthcare, which usually has a needs-based focus, the PSI-WPI caused a shift towards providing resources to patients with higher work ability and (perhaps) lower needs for healthcare (though patients with higher healthcare needs will receive other resources, adapted to their needs and capabilities). Within the general healthcare, the view was that patients with similar needs should be given similar resources. Within the PSI-WPI, the employee must have been capable of participating in the intervention and must also have accepted first-line manager involvement. Consequently, a sub-group was generated within the population of people with CMDs—that is, a sub-group of people who met these two requirements which conflicted with healthcare’s requirement for equal access. One rehabilitation coordinator said “*Now they were sifted out, those who didn’t want healthcare to contact their manager”* (rehabilitation coordinator 1).

### Sub-theme: The PSI-WPI challenged the professional roles of first-line managers and rehabilitation coordinators

The introduction of the PSI-WPI caused a shift in the role of the first-line manager and rehabilitation coordinator in relation to the employee’s privacy and support for the employee’s autonomy.

The PSI-WPI expanded the role of the first-line managers by having them participate in a problem-inventory, and the three-party meeting. A conflict of aims could have arisen when the first-line managers had to find the best solution for the employee on SA, the company, and other employees. Even if the responsibility for and arrangement of workplace accommodations was on the employer and regulated by law (Ministry of Labour, 1977), the possibility to offer work accommodations could depend on factors outside the employer’s and first-line manager’s control, such as the specific characteristics of the workplace. One first-line manager explained: “*As a manager, we can do certain parts, but we can’t do everything and we can’t adapt as much as the employee want without them also having to understand that somewhere, what shall I say, our responsibility ends somewhere. We can adapt to a certain level, but they also need to take responsibility. To find these boundaries for who should do what*” (First-line manager 1).

During the PSI-WPI, the rehabilitation coordinators were expected to act in a neutral way, i.e., to support both the employee and their first-line manager and to host three-party meetings. However, in the role of a health care professional, the norm was to be the patient’s advocate. The participating rehabilitation coordinators were trained registered nurses, physiotherapists, or occupational therapists. All but one had their rehabilitation coordinator role split into part-time appointments, working as a health care professional and rehabilitation coordinator on different days of the week. This divided time made the shift between the roles more difficult, and rehabilitation coordinators reported sometimes lacking tools for how to adapt and keep these roles apart, especially when meeting the employee in both roles. One rehabilitation coordinator explained, “*It’s easy, as a new rehab coordinator, to assume that you’re sort of the patient’s advocate, however the role is to coordinate. You shouldn’t, what should I say, choose a side, it should … it should be from two sides, that’s really important, and you become aware in this situation that you get input from both managers and employees, and that there are often two sides to the story”* (Rehabilitation coordinator 2). Various measures were used by the rehabilitation coordinators to adapt to their role such as explaining their role at the meetings with the employee and first-line manager. Still, some rehabilitation coordinators argued that, even when serving as a rehabilitation coordinator, the role was to be the patient’s advocate so as to enable a trustful relationship with the employee and ensure good collaboration. Even if employees and first-line managers agreed that the role of the rehabilitation coordinator should be neutral they also reported disappointment when the rehabilitation coordinator did not support their case during the three-party meeting.

During the PSI-WPI, information related to the employee’s health, symptoms, work-life and private life were discussed and sometimes transferred between the stakeholders, both with and without the employee present. This process required the rehabilitation coordinator to handle and share information more extensively, implying a greater risk to patient privacy than when that rehabilitation coordinator was in the role of a health care professional. Likewise, the first-line manager needed to handle health-related information, which was not normally part of their role, and which brought with it high demands for securing privacy. The rehabilitation coordinators handled this need by asking the employee, before the three-party meeting, what information should be shared with the first-line manager. The sharing of information could also go the other way; that is, the first-line manager could reveal workplace-related information that the employee had not shared with the rehabilitation coordinator. Consequently, the PSI-WPI involved a more extensive sharing of information than the usual strict regulation of information sharing within healthcare.

The role of the rehabilitation coordinator during the PSI-WPI meant supporting the employee’s autonomy by identifying problems and finding solutions to enable that individual to return to work, which may have been different from their usual role as a health care professional, in which recommending treatments was more common. For the first-line manager, the expectation for an employee’s autonomy was decreased and meant, for example, arranging accommodated work tasks and taking health-related problems into account. Yet, despite expectations that the employee would be autonomous, the rehabilitation coordinator and first-line manager may also have needed to prevent the employee from taking on tasks in conflict with their current work ability and have guided them forward during the return to work process.

### Sub-theme: The PSI-WPI introduced a need to juggle the employee and patient roles

The PSI-WPI challenged the employee’s privacy in that it decreased the individual’s ability to control their personal information. Involving the first-line manager introduced a need for the employee to juggle the roles of an employee and patient.

The sharing of information among the stakeholders in the PSI-WPI was expected to increase the stakeholders’ understanding of problems and solutions in relation to the employee’s return to work. The information was, with the consent of the employee, transferred between stakeholders (i.e., from health care to the workplace, and vice versa) with or without the employee present on the information sharing occasion, for example, during the problem-inventory between the first-line manager and rehabilitation coordinator, and to some extent in the three-party meeting. This information sharing took place outside of the employee’s control which would normally (i.e., not during PSI-WPI) not be the case.

The intention of the PSI-WPI was that on the one hand, sharing information between the workplace and healthcare could enhance the employee’s possibility of receiving support at the workplace with, for example, health-related issues and following the return to work plan. A joint understanding among stakeholders was expected to help the employee and first-line manager to understand the expectations put upon them. On the other hand, employees experienced that information sharing could have a downside. Revealing health-related or sensitive information, that potentially affected their work ability to the first-line manager could influence future opportunities at the workplace and thus the employee faced disclosure dilemmas and needed to navigate the right amount of sharing. One employee described her thoughts on the disclosure process like this: *“I think about it this way, right at the beginning when I started my sickness absence period, my doctor wrote a certificate to my manager that didn’t say anything. You can choose not to have everything included. And… well…afterwards we decided to change it so that at least she gets the information properly, I think that you need to so that you can create a good relationship with the persons involved, as the manager and so on, it’s probably an advantage if they know everything, generally speaking. However, if you don’t have a good relationship, it could be at your own demise” (Employee 1)*

Some employees and first-line managers considered it helpful to inform a sick-listed employee’s colleagues of the situation to ensure that the colleagues understood the expectations and assignments during the accommodated work period, and to prevent pressure and unrealistic expectations. However, employees feared stigmatization related to sharing health-related information with colleagues and needed to balance potential beneficial effects with negative ones.

Before their three-party meeting, employees were encouraged to discuss what information they were prepared to disclose to their first-line manager. During that discussion, an employee needed to consider their role as a patient versus their role as an employee. In the patient role, the focus was on expressing symptoms and needs with the goal of receiving treatment, and not considering whether information would be shared outside the patient/health care professional encounter. In the role of an employee on sickness absence, the goal was to return to work and portraying a soon-to-be capable employee, requiring the employee to be more on guard. The next step of the PSI-WPI was the three-party meeting at which an employee usually wanted to be in the employee role and be more restrictive about sharing information about compromised work ability than they might be in the patient role. One employee said “*When I met her, the rehabilitation coordinator—for the first time I felt at ease. Like she really understood me. So that’s why I was also a little surprised, during the meeting with my manager when it became like … that she didn’t take my side or stand up for me at all*” (Employee 2). This quote was an example of challenges both in regard to which role the employee took, and in regard to the expectations they had on the rehabilitation coordinator.

## Discussion

The aim of this study was to explore ethical challenges potentially arising from a PSI-WPI in primary health care with first-line manager involvement for employees on sickness absence due to common mental disorders. Our main results showed that the PSI-WPI, provided as an individual-level intervention with collaboration between the workplace and healthcare, resulted in ethical challenges at organizational and individual levels. The different, and in some cases conflicting value logics, values, and goals of the organizations introduced several challenges regarding: i) rehabilitation coordinators and first-line managers’ professional roles ii) effects on third parties iii) fair distribution of resources and iv) a need for the employee to juggle the roles of an employee and patient. The most prominent ethical challenge on the individual level was the sharing of health- and work-related information among the stakeholders (i.e., employee, first-line manager and rehabilitation coordinator). Information sharing about the employee sometimes took place without the employee being present, and at these times, the employee had limited control over information sharing. This lack of control deviated from the usual handling of information within healthcare which is highly restricted by secrecy legislations and therefore introduces disclosure dilemmas on part of the employee.

Our results indicated that these perceived disclosure dilemmas highlight a responsibility about deciding which, and how much, information an individual wants and needs to share with the workplace and healthcare. Beneficial information sharing among the employee, workplace and healthcare has been shown to enhance stakeholders’ abilities to support the employee during their return to work process (Toth & Dewa, 2014). But disclosure may come at the cost of the employee fearing stigma and decreased opportunities for future promotions (Brohan et al., 2012; Toth et al., 2021). Even so, most employees reported positive reactions from their first-line managers when they disclosed, despite feeling negative emotions and stress about the disclosure beforehand (Toth et al., 2021). In turn, managers have reported that learning of their employees’ problems is vital for being able to provide sufficient support (Porter et al., 2019). To support the employee in the disclosure process, a preparatory discussion between the employee and rehabilitation coordinator has been recommended to help the employee keep a focus on problems that affect work ability (Holmlund et al., 2022). In the case of PSI-WPI, the early phase of the intervention was suitable for such discussions, for example, alongside the inventory of problems and solutions with the employee and rehabilitation coordinator present. The rehabilitation coordinator can help the employee decide whether to share, and how much information needs to be revealed about their common mental disorder-related symptoms as well as the potential benefits and harms that may follow. In line with previous research (Holmlund et al., 2022) we recommend discussing disclosure early in the return to work process, and providing the employee an opportunity to think through and prepare themselves for handling potential disclosure dilemmas while being supported by the rehabilitation coordinator.

Previous research suggests that supporting employees in their return to work process through work-directed interventions entails a complex interplay among various stakeholders, who all may have different goals or be governed by different laws and regulations affecting the return to work process (Holmlund et al., 2022; Loisel et al., 2013; Ståhl et al., 2014; Young et al., 2005). Our results exemplified how this complex interplay on the organizational level between the workplaces and healthcare’s different and sometimes conflicting value logics also manifested the individual level. Through the lens of the Swedish context, where the safety, health, and rehabilitative responsibilities of an employee is highly regulated by law (Ministry of Labour, 1977), the employer’s value logics (including the employer’s productivity goals) were still emphasized. In Sweden, return to work is facilitated by the sickness insurance system and in 2008, the “rehabilitation chain” was introduced by the Swedish Social Insurance Agency. The rehabilitation chain starts on day 15 of sickness absence and assesses the employee’s work ability at consecutive time points. From day 15 to day 180, the employees work ability is assessed in relation to any work tasks at the current workplace while from day 181 to day 365 the employee’s work ability is assessed in relation to work tasks suitable for the employee’s work ability at any workplace (The National Board of Health and Welfare, 2023).

For the employees, the influence of the PSI-WPI on their role involved juggling the employee and patient roles, i.e., conveying the impression of a soon-to-be capable employee versus expressing symptoms in order to receive treatment. Previous research has found that employees on sickness absence often take on a patient role to reinforce evidence of their illness, but when they see themselves as recovering, the employee role becomes more prominent. Thus, supporting the employee in maintaining the employee role, adapted to their current ability to work, has been reported to be important for the return to work process (Li & Wolbring, 2019; Millward et al., 2005).

For first-line managers, the PSI-WPI influenced their role by requiring them to handle sensitive information, which could further reinforce the conflict among responsibilities that first-line managers must handle in everyday work. A structural organizational problem related to PSI-WPI and return to work issues in general is that first-line managers need to ensure the company’s productivity while also being responsible for employees’ rehabilitation, including work accommodations. Work accommodations potentially affect both productivity and the work burden of other employees without financial compensation to the employer or colleagues who take on a larger work burden, which has also been observed elsewhere (Porter et al., 2019). Similar observations regarding work accommodations have been reported among managers. They find it difficult to balance the contradictory needs and demands from the employee on sickness absence due to a mental disorder and those of his/her colleagues (Suter et al., 2022).

For rehabilitation coordinators, the influence of the PSI-WPI on their role highlighted the difficulties of separating the role of a health care professional from the role of a rehabilitation coordinator (i.e., acting as a patient advocate versus a neutral coordinator), and the need to consider not only the interest of the employee, but also the first-line manager and the workplace. All rehabilitation coordinators in our sample were trained nurses, occupational therapists, or physiotherapists which are professions with norms and work ethics based on being a patient advocate. Adapting to these two different roles may introduce conflicting norms and choices, an ambiguity which also makes it more difficult for the rehabilitation coordinator to provide coordinator services, as has been reported elsewhere (James et al., 2011).

Our suggestions for how to handle the influence of the PSI-WPI on stakeholders’ roles are for the rehabilitation coordinator to be supported with a discussion around their own norms and expectations, and that the norms and expectations of other stakeholders’ and their roles be clarified during their training. Practicing thinking through these issues can give rehabilitation coordinator’s an opportunity to define the norms relating to each role and an understanding of how to act in situations when roles may be conflicting. The rehabilitation coordinator coordinates the return to work process and should initiate a discussion with the employee and the first-line manager about roles, responsibilities, and expectations, and support involved stakeholders with knowledge about return to work procedures, regulations, and recommendations. Employers have a legal responsibility to ensure a healthy work environment (Ministry of Labour, 1977) and provide for the rehabilitative needs of their employees. Despite this regulated responsibility, research on the knowledge of employers about the return to work process, especially for employees who were on sickness absence for reasons related to mental illness, has been reported to be insufficient and employers has pointed out a need for support with return to work procedures (Porter et al., 2019), which is in line with our results. This lack of knowledge has also been pointed out by the Organisation for Economic Co-operation and Development which recommend that employers receive training to detect mental health problems at the workplace and integrate mental health treatment at work (OECD, 2021).

Our results may be perceived as meaning that the PSI-WPI sets aside the healthcare values of fairness and equal distribution of resources, because it prioritized patients with less need for healthcare and a greater ability to work. Among persons with common mental disorders, the majority are employed (OECD, 2012). Thus, the participants in the PSI-WPI may be considered to have a high chance of returning to work. However, for the large group of employees on sickness absence due to common mental disorders, the potential benefit of a PSI-WPI in terms of reduced individual suffering and possible shortening of sickness absence could be motivated by the potential cost-effectiveness and use of healthcare resources.

In our study, first-line managers and rehabilitation coordinators discussed that complying with employee needs and autonomy may conflict with a request for a prolonged sickness absence. Adverse effects of sickness absence due to common mental disorders have been reported to potentially result in recurring sickness absence, going on disability pension, and unemployment (Mather et al., 2019). The benefits of employment have been well described (Modini et al., 2016; van der Noordt et al., 2014), and it may not always be beneficial to support a request for sickness absence. Instead, it´s better to support employees with problem-solving and accommodating work tasks, as intended in the PSI-WPI. We recommend that the rehabilitation coordinator, first-line manager and employee jointly discuss the beneficial effects of work as well as the potentially adverse effects of sickness absence early in the absence period.

Our main take-home message about how to handle the ethical challenges regarding conflicting value logics, disclosure dilemmas, juggling of roles, and shifts in professional roles is that the roles and responsibilities of involved stakeholders in the return to work process need to be clear. This recommendation was given by Holmlund et al. in their evaluation of ethical aspects of coordinating return to work (Holmlund et al., 2022), and the importance of clarity about stakeholders’ roles has been further exemplified in the return to work literature (Corbière et al., 2020). Thus, defining stakeholders’ roles and providing the employee and first-line manager with sufficient information on return to work processes may avoid some of the challenges reported. Additionally, though the results of this evaluation are specific to the PSI-WPI context, the ethical challenges and the suggestions for how to minimize them may be considered when planning similar return to work interventions.

## Strengths and limitations

A strength of our study is the inclusion of three stakeholders (employees, first-line managers, and rehabilitation coordinators) which allowed us to get a good understanding of the challenges related to the individual, the workplace, and healthcare. The interviews conducted by two researchers with different educations and experiences ensured a wide competence, and the support of the principal investigator ensured a consistent approach to data and analyses. To avoid social response bias, participants were informed that the interviewers were not part of the intervention design. Most interviews were conducted by phone due to the COVID-19 pandemic, and telephone interviews have been reported to be preferable to in-person interviews because of the increased flexibility and better balancing of power relations (Azad et al., 2021). Trustworthiness was strengthened by informing participants about the purpose of the study, the confidentiality procedures, and by beginning each interview with an explanation of the intervention. Both the method of analysis and results can readily be transferred to other return to work interventions in countries with similar return to work policies.

The study also had limitations. The strategic sampling initially planned was not successful, with only 13 out of 72 employees agreeing to participate, and the sample of employees and rehabilitation coordinators included only females. Recruitment for research studies is difficult (Foldal et al., 2020), and may have been hampered further by the employees’ common mental disorder diagnoses. However, no differences in symptoms at baseline were found between the interviewed employees and the rest of the employees in the PROSA sample. Employees and first-line managers were interviewed twelve months post-inclusion in the PROSA trial due to allocation blinding, which may have introduced recall bias.

## Conclusions

Different organizational value logics, different values, and different goals can introduce ethical challenges, especially in regard to sharing of information among stakeholders. These challenges led to disclosure dilemmas on behalf of the employee, and uncertainty about roles and expectations for the first-line managers and rehabilitation coordinators. Both employees and rehabilitation coordinators had to negotiate what it meant to be a patient and employee. We recommend that defining stakeholders’ roles be made a priority. The ethical challenges and suggested measures for minimizing them, should be considered when planning return to work interventions involving several stakeholders.

## Data Availability

The data is not publicly available due to containing information that could compromise the privacy of the study participants. Reasonable inquiries about access may be sent to Karolinska Institutet, Institute of Environmental Medicine, Unit of Intervention and Implementation Research for Worker Health, Box 210, 171 77 Stockholm or by contacting the Research and Data Office at Karolinska Institutet: rdo@ki.se. The Swedish Ethical Review Authority will then be contacted for permission.

## Abbreviation

PSI-WPI: problem-solving intervention with workplace involvement

## Appendices

### Appendix 1, Interview guides

#### Interview guide for employees

The PROSA-intervention consists of 1) the employee meeting the rehabilitation coordinator at the primary health care to map his/her situation and problems and solutions for return to work, 2) a three-party meeting with the first-line manager, you as employee, and the coordinator, and 3) a follow-up meeting together with the coordinator, and with your first-line manager possibly participating, either in person or by phone. You will receive questions and we will discuss ethical considerations and/or challenges related to participating in the intervention.

**Autonomy**

Main question: How do you feel about being involved in the decisions that were made regarding your return to work process?

- What opportunities did you have to be involved in the decisions about your rehabilitation and return to work?
- To what extent were you motivated to act independently?
- In what way did the PROSA intervention enable you to make decisions about your return to work?
- In what way did the PROSA intervention prevent you from making decisions about your return to work?

**Integrity**

Main question: How do you see the sharing of sensitive information (illness, private situation, etc.)?

- What personal information is important to share in the return to work process?
- What kind of information might be perceived as sensitive to share?
- What reasons can there be for restricting information sharing?
- In which situations?
- Can you give examples of how such restrictions can be justified?
- Can you give examples of situations where you do not want to share information with other stakeholders (e.g., employer, the Social Insurance Agency)?
- How has that been handled?

**Equality**

Main question: What demands for self-care (alternatively to take care of one’s own health) did the PROSA intervention put on you when you were on sickness absence due to a common mental disorder.

- Are there demands that became difficult for you to handle?
- What do you think, if the PROSA intervention is introduced in primary health care, which sick listed employees should be offered the intervention?

Should the intervention:

- Be offered to everyone who is on sickness absence due to a common mental disorder?
- Be offered to those who need extra support from primary health care?

What do you think, will the intervention:

- Lead to more people with common mental disorders returning to work?
- Favour certain groups (if so, which ones)?
- Benefit those who need extra support from healthcare (if so, why)?

**Justice**

Main question: How do you see the possibility of the PROSA intervention being given as a routine at primary health care (in relation to everything else that is done at the primary health care)?

- How do you think about the fact that resource limitations in healthcare can affect the application of the PROSA intervention?
- If so, what priority should the PROSA intervention be given?

**Professional values**

Main question: What expectation can one have that primary health care will represent you as an employee?

- What do you think, is the PROSA intervention “in line with” primary health care’s mission (i.e., should primary health care work in collaboration with first-line managers)?
- Based on your experiences, who did the rehabilitation coordinator represent, for example, when meeting with you and your first-line manager?

Are there other ethical challenges of the PROSA intervention that you consider as important, but that we have not discussed?

#### Interview guide for first-line managers

The PROSA-intervention consists of 1) the employee meeting the rehabilitation coordinator at the primary health care to map his/her situation and problems and solutions for return to work, 2) a three-party meeting with your employee, you as first-line manager and the coordinator, and 3) a follow-up, in which you as first-line manager possibly have participated, either in person or by phone. You will receive questions and we will discuss ethical considerations and/or challenges related to participating in the intervention.

**Autonomy**

Main question: How do you feel about employees being involved in the decisions made regarding return to work?

- What opportunities do you think the employee himself has to make decisions about the return to work process?
- To what extent is the employee motivated to act independently?
- In what way does the PROSA intervention enable the employee to make decisions about returning to work?
- In what way does the PROSA intervention prevent the employee from making decisions about returning to work?

**Integrity**

Main question: How do you see the fact that the employee may have to share sensitive information (Illness, private situation, etc.)?

- What personal information do you think is important to share, in the return to work process?
- What kind of information do you think could be perceived as sensitive to share?
- What reasons can there be for restricting information sharing?
- In what situations?
- Can you give examples of how such restrictions are justified?
- Can you give examples of situations where you did not want to share information with other stakeholders (e.g. primary health care, the Social Insurance Agency)?
- How has that been handled?

**Equality**

Main question: What demands for self-care (alternatively to take care of one’s own health) does the PROSA intervention put on employees who have suffered from common mental disorders?

- Are there demands that are difficult to handle for employees with, for example, depressive symptoms or anxiety?
- What do you think, if the intervention is introduced in primary health care, which patients should be offered the intervention?

Should the intervention:

- Be offered to everyone who is on sickness absence due to common mental disorder?
- Be offered to those who need extra support from primary health care?
- What do you think, will the intervention:
- Lead to more people with common mental disorders returning to work?
- Favour certain groups (if so, which ones)?
- Benefit those who need extra support from healthcare?

**Justice**

Main question: How do you see the possibility of the PROSA intervention being given as a routine at primary health care (in relation to everything else that is done at the primary health care)?

- How do you think about the fact that resource limitations in healthcare can affect the application of the PROSA intervention?
- If so, what priority should the intervention be given?

**Professional values**

Main question: What expectation can one have as an employer that primary health care should be neutral?

- Based on your experiences, who does the rehabilitation coordinator represent?

Are there other ethical challenges of the three-party meeting or the PROSA intervention that we haven’t touched on that you would like to include?

#### Interview guide for rehabilitation coordinators

The PROSA intervention consists of 1) a meeting between the employee and the coordinator at the primary health care to map his/her situation and problems and solutions for return to work, 2) a three-party meeting between the coordinator, the employee and first-line manager and 3) a follow-up, either by phone or in person between the coordinator and the employee (and potentially with the first-line manager). You will receive questions and we will discuss ethical considerations and/or challenges related to providing the intervention.

**Autonomy**

Main question: How do you feel about the employee being involved in the decisions made regarding

return to work?

- What opportunities does the patient have to make their own decisions about the return to work process?
- To what extent is the patient motivated to act independently?
- In what way does the PROSA intervention enable the employee to make decisions about returning to work?
- In what way does the PROSA intervention prevent the employee from making decisions about returning to work?

**Integrity**

Main question: How do you see the sharing of sensitive information during the PROSA intervention (Illness, private situation, etc.)?

- What personal information do you think is important to share in the return to work process?
- What kind of information do you think might be perceived as sensitive to share?
- What reasons can there be for restricting information sharing?
- In what situations?
- Can you give examples of how such restrictions are justified?
- Can you give examples of situations where the patient did not want to share information with other stakeholders?
- How have you dealt with it?

**Equality**

Main question: What demands on self-care (alternatively to take care of one’s own health) does the PROSA intervention put on employees with common mental disorders?

- Are there demands that are difficult to handle for employees with, for example, depressive symptoms or anxiety?
- What do you think, if the intervention is introduced in primary health care, which patients should be offered the intervention?

Should the intervention:

- Be offered to everyone who is on sickness absence due to common mental disorders?
- Be offered to those who need extra support from primary health care?

What do you think, will the intervention:

- Lead to more people with common mental disorders returning to work?
- Favor certain groups (if so, which ones)?
- Benefit those who need extra support from healthcare?

**Justice**

Main question: How do you see the possibility of introducing the PROSA intervention as a routine at the primary health care (in relation to everything else that is done at the primary health care)?

- How do you think about resource limitations that may affect the application of the PROSA intervention?
- If so, what priority should the intervention be given?

**Professional values**

Main question: How do you think about the PROSA intervention—does it match the mission as a rehabilitation coordinator?

- How do you see that the PROSA intervention is aimed at returning to work?
- When performing the PROSA intervention, who are you representing (employee/manager)?

Are there other challenges of the PROSA intervention that we haven’t touched on that you’d like to include?
